# Control and Elimination of Schistosomiasis as a Public Health Problem: Thresholds Fail to Differentiate Schistosomiasis Morbidity Prevalence in Children

**DOI:** 10.1093/ofid/ofab179

**Published:** 2021-04-15

**Authors:** Ryan E Wiegand, W Evan Secor, Fiona M Fleming, Michael D French, Charles H King, Susan P Montgomery, Darin Evans, Jürg Utzinger, Penelope Vounatsou, Sake J de Vlas

**Affiliations:** 1 Division of Parasitic Diseases and Malaria, Centers for Disease Control and Prevention, Atlanta, Georgia, USA; 2 Swiss Tropical and Public Health Institute, Basel, Switzerland; 3 University of Basel, Basel, Switzerland; 4 SCI Foundation, London, United Kingdom; 5 RTI International, Washington, DC, USA; 6 Center for Global Health and Diseases, Case Western Reserve University, Cleveland, Ohio, USA; 7 United States Agency for International Development, Washington, DC, USA; 8 Department of Public Health, Erasmus MC, University Medical Center Rotterdam, Rotterdam, the Netherlands

**Keywords:** control, elimination, mass drug administration, morbidity, preventive chemotherapy, schistosomiasis, ultrasound

## Abstract

**Background:**

Current World Health Organization guidelines utilize prevalence of heavy-intensity infections (PHIs), that is, ≥50 eggs per 10 mL of urine for *Schistosoma haematobium* and ≥400 eggs per gram of stool for *S. mansoni*, to determine whether a targeted area has controlled schistosomiasis morbidity or eliminated schistosomiasis as a public health problem. The relationship between these PHI categories and morbidity is not well understood.

**Methods:**

School-age participants enrolled in schistosomiasis monitoring and evaluation cohorts from 2003 to 2008 in Burkina Faso, Mali, Niger, Tanzania, Uganda, and Zambia were surveyed for infection and morbidity at baseline and after 1 and 2 rounds of preventive chemotherapy. Logistic regression was used to compare morbidity prevalence among participants based on their school’s PHI category.

**Results:**

Microhematuria levels were associated with the *S. haematobium* PHI categories at all 3 time points. For any other *S. haematobium* or *S. mansoni* morbidity that was measured, PHI categories did not differentiate morbidity prevalence levels consistently.

**Conclusions:**

These analyses suggest that current PHI categorizations do not differentiate the prevalence of standard morbidity markers. A reevaluation of the criteria for schistosomiasis control is warranted.

Schistosomiasis was estimated to have caused 1.1 million years lived with disability in 2017, roughly a third of the disability years associated with tuberculosis and about three-quarters that of malaria [1]. The disease is caused by *Schistosoma* spp. trematode worms, which can survive in the human body for years to decades. Morbidity mainly occurs due to inflammation and scarring from parasite eggs that become lodged in the capillaries of either the urogenital system as a result of infection with *Schistosoma haematobium* or the liver and intestines from infection with *S. mansoni* and other schistosome species [2, 3]. Urogenital schistosomiasis causes hematuria in children [4]. More chronic infections are characterized by fibrosis of the urinary tract [5], obstruction of urine flow [5], female [6] and male [7] genital schistosomiasis, and, rarely, bladder cancer [8]. Intestinal species commonly cause abdominal pain, diarrhea, and blood loss in the stool [4]. Chronic infections can lead to scar tissue in the bowels, hepatosplenic disease, periportal fibrosis, and collateral circulation [3], which can lead to exsanguination.

The World Health Organization (WHO) has stated that the primary goal of schistosomiasis control programs is to reduce morbidity [9–11]. Current WHO guidelines [12] use heavy-intensity infections (≥50 *S. haematobium* eggs per 10 mL of urine or ≥400 *S. mansoni* eggs per gram of stool [10]) to categorize a target population’s status. Target populations with a prevalence of heavy-intensity infections (PHI) <5% are classified as having controlled schistosomiasis morbidity, and when a target population has <1% PHI, that population has eliminated schistosomiasis as a public health problem. These PHI thresholds are used despite treatment frequency being determined by the prevalence of any infection [12]. The selection of the PHI thresholds was based on correlations between infection intensity and severe pathology reported from limited data that were collected before 1990 [13, 14]. Those studies demonstrated a statistically significant difference between noninfected and heavily infected participants, but not between noninfected and lightly infected participants. These findings, in an era in which treatment was limited and expensive, led to the development of program guidance focused on reducing heavy-intensity infections associated with the types of severe morbidity that are more common in older individuals [15]. More recent studies have found that less clinically severe manifestations, which can occur even with light infections and are common in school-age children, have a greater impact on population-level disability-adjusted life-years lost, that is, an estimate of the years of life lost due to poor health, disability, or early death, compared with the most severe pathologies [2]. Furthermore, the earlier guidelines failed to consider the limitations of a single stool or urine sample from study participants. As a result, lighter infections and their association with morbidity were likely underestimated [16].

Our search of the literature did not uncover a formal assessment of the relationship between morbidity and the 1% and 5% PHI thresholds. Therefore, in response to a recent call to update the WHO guidelines for monitoring morbidity control to evidence-based targets [17, 18], we evaluated the relationship between aggregate morbidity and the WHO heavy-intensity infection thresholds used to define control and elimination of schistosomiasis as a public health problem. Data from Schistosomiasis Control Initiative (SCI)–supported preventive chemotherapy programs collected between 2003 and 2008 in different African countries [19] were used to test for differences in morbidity levels in children between PHI categories before and after initiation of the mass drug administration (MDA) of praziquantel [20].

## METHODS

### Study Design and Data Collection

Data were collected as part of national programs for schistosomiasis and soil-transmitted helminthiasis control from 2003 to 2008 in 6 African countries (Burkina Faso, Mali, Niger, Tanzania, Uganda, and Zambia) supported by the SCI [19, 21–24]. Each country developed a control program for national scale-up, which involved prevalence mapping to determine at-risk populations, MDA of praziquantel (for schistosomiasis) and albendazole (for soil-transmitted helminthiasis) to school-age children and, in some countries, adults in high-risk communities, and a monitoring and evaluation framework. Each country implemented praziquantel distribution to target populations at the frequency recommended by WHO guidance [25]. Countries maintained 2 types of monitoring and evaluation cohorts. The first was a cohort of children aged 6–12 years enrolled in primary school. These children were ascertained for infection and morbidity indicators at baseline and evaluated before receiving treatment. They were then followed and treated annually for 2 more years, with some exceptions: Zambia, where there was only 1 year of follow-up; Mali, where some schools were treated twice between baseline and follow-up 1 and others were treated annually but not followed up at follow-up 1; and Tanzania, where schools were not treated between follow-up 1 and follow-up 2. At the point when a school was not treated once annually, it was omitted from that survey and subsequent surveys. The second was a community cohort of mostly adults but included persons 4–88 years old. A random sample of the community was ascertained for infection and morbidity indicators and evaluated before treatment at each round. The small sample of children in this cohort were likely absent from school that day. Each community was followed up for the same number of years as the longitudinal cohort. In both cohorts, individuals feeling unwell or <94 cm in height were ineligible for praziquantel treatment and excluded. Communities were randomly selected via stratified sampling. Strata were based on endemicity levels to provide a wide representation of different areas. For these analyses, we limited our analyses to 6–15-year-olds in either cohort, with at least 30 participants in a school possessing infection and morbidity data.

### Infection Data

A single urine filtration to evaluate *S. haematobium* was used in Burkina Faso, Tanzania, and Zambia. Two filtrations from the same urine sample were used in Niger, and 2 different urine samples from consecutive days were used in Mali. Uganda did not collect *S. haematobium* infection data. Urine filtration was performed by passing ~10 mL of urine through a filter that was then stained and microscopically examined for eggs. An individual’s intensity was calculated as the arithmetic mean number of eggs per 10 mL of urine across all available samples. Heavy-intensity infection was defined as ≥50 eggs per 10 mL of urine [10].

For *S. mansoni*, the Kato-Katz technique with thick fecal smears microscopically examined for eggs was used. A single stool, with 2 different slides (each 41.7 mg) examined per stool, was used in Burkina Faso, Mali, Niger, and at baseline in Uganda. Tanzania, follow-up surveys in Uganda, and Zambia used 2 stools from consecutive days with 2 slides each per stool. Individual intensity was computed by multiplying the number of eggs per slide by 24 to calculate the eggs per gram of stool (EPG), then taking the arithmetic mean of all available samples measured for that child. Participants with ≥400 EPG were defined as having a heavy-intensity infection [10].

### Morbidity Data

Ultrasound exams were performed according to the Niamey protocol, which has standardized examinations and reporting practices for *S. haematobium* and *S. mansoni* ultrasonic evaluations [26]. *S. haematobium* ultrasounds were done in Mali, Niger, Tanzania, and Zambia, though in Zambia results were rare at baseline and were confined to follow-up 1. Participants were evaluated by ultrasound for bladder abnormalities, defined as distorted bladder shape, irregular bladder wall, bladder wall masses, pseudopolyps, or thickening of the bladder wall, and upper urinary tract abnormalities were defined as a dilated renal pelvis (left or right) or dilated ureter (left or right). For analyses, we deviated from the scoring system used in the Niamey protocol and categorized abnormalities in 2 ways. The first approach looked across indicators and coded a participant positive who had at least 1 ultrasound abnormality and coded a participant negative if the participant had no ultrasound abnormalities. Only participants with data for all ultrasound indicators were included in those analyses to ensure that each participant possessed the same chance of possessing a positive ultrasound indicator. The second approach summed up the number of ultrasound abnormalities per person among participants with any available ultrasound data, which allowed all ultrasound data to be included. *S. mansoni* ultrasounds were performed in Mali, Niger, Tanzania, Uganda, and Zambia, with Zambian results only in follow-up 1 as noted above. Participants were evaluated for enlarged portal vein and liver image patterns indicative of schistosomiasis-associated fibrosis. Under the Niamey protocol, image pattern A is considered normal, image pattern B is indeterminate, and image patterns C and above are considered indicative of schistosomiasis-associated fibrosis, where these correspond to small patches of rings and pipe stems throughout the liver parenchyma (pattern C), fibrosis around the main stem of the liver (D), more substantial fibrotic patches around the main stem (E), and extensive fibrosis throughout the parenchyma (F). As the Niamey protocol scores a person with image pattern B with 1 point and, at a minimum, does not exclude the possibility of periportal fibrosis, we assessed the frequency of both image patterns C–F and B–F as evidence of periportal thickening.

Additional morbidities were collected in all 6 countries. These included microhematuria assessed with Hemastix dipsticks [27] and self-reported painful urination [28] for *S. haematobium* and 2 measures confirmed by laboratorians (diarrhea and blood in stool) and 2 self-reported measures from the last 2 weeks (diarrhea and abdominal pain [29, 30]) for *S. mansoni*.

### Data Analysis

Data from all countries were harmonized and pooled for analyses in R, version 4.0.3 [31]. The PHI was calculated per school at each survey, and schools were split into 3 categories of PHI (PHI <1%; PHI ≥1% and <5%; and PHI ≥5%). Individuals were analyzed based on the school’s category.

Prevalence estimates were calculated in the survey package [32] after accounting for clustering at the school level and countries as strata. These analyses used the 5% level of significance. Bayesian logistic regression models were fit via Markov chain Monte Carlo (MCMC) methods in JAGS [33] for each indicator except for the sums of *S. haematobium* ultrasound indicators, for which binomial models were used. Survey year, heavy-intensity infection category, and their interaction were included as fixed effects. Indicator variables for countries were added to control for differences between control programs. Random intercepts were included for schools and individuals to account for multiple observations sampled from the same school or individual, respectively. All comparisons are reported as odds ratios (ORs) with 95% Bayesian credible intervals (BCIs) from the posterior distributions. Full details of the models as well as results from the binomial models, individual ultrasound indicators, and other laboratory and self-reported morbidities are included in the Supplementary Data.

### Patient Consent Statement

The Imperial College Research Ethics Committee (ICREC_8_2_2, EC No. 03.36, R&D No. 03/SB/003E) and the ethical review boards of the Ministries of Health of the 6 countries provided ethical approval for use of these data. The US Centers for Disease Control and Prevention was determined to be a nonengaged research partner. In all countries, meetings were held with teachers and parents to inform them about participation in these programs. In Mali, verbal consent from community leaders was obtained, as this was the most accepted form of consent at the time. In all other countries, written informed consent was obtained from head teachers at each school. Agreements from parents or guardians were obtained as well as assent from children.

### Role of the Funding Sources

The sponsors of this study played no role in the design, collection, analysis, interpretation, or composition of this report. All authors contributed to the decision to submit for publication.

## RESULTS

### Schistosoma haematobium

**Figure 1.**
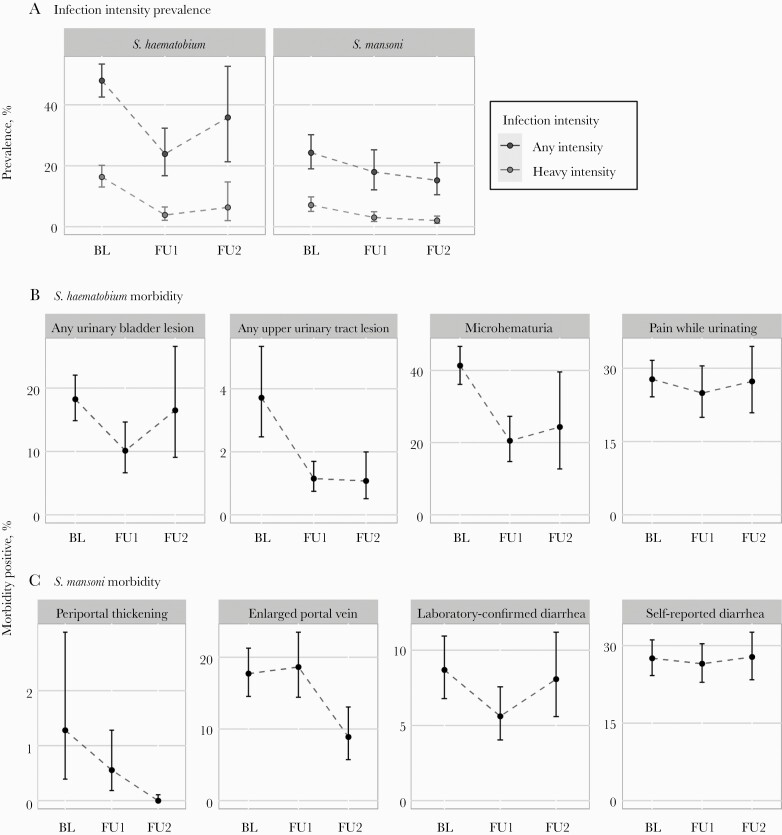
Line graphs of the prevalence of 6–15 year-old school children who were *Schistosoma* infection and heavy-intensity positive (row A), *S. haematobium* morbidity positive (row B), and *S. mansoni* morbidity positive (row C) at each survey year (BL = baseline, FU1 = follow-up 1, FU2 = follow-up 2). *S. haematobium* infections were assessed by urine filtration and *S. mansoni* infections by Kato-Katz. Plots of individual ultrasound indicators for *S. haematobium* and additional morbidity indicators for both species are included in Supplementary Figures 1 and 2.

The numbers of schools and participants that contributed *S. haematobium* morbidity data ranged from 7 to 59 schools and 861 to 7766 participants per PHI category in a survey (Supplementary Table 1). Prevalence of infection and PHI decreased at follow-up surveys as compared with baseline (Figure 1A, left). Overall decreases in prevalence were also experienced for most morbidities (Figure 1B; Supplementary Figure 1).

Modeling results found that participants in schools with PHI <1% and 1–5% PHI had lower odds of most morbidities as compared with participants from schools with PHI ≥5% (Table 1). For participants in schools with PHI <1% compared with participants in schools with PHI ≥5%, odds were lower at each survey for any bladder lesion, microhematuria, and pain while urinating. For participants in schools with 1–5% PHI compared with participants from schools with PHI ≥5%, odds were lower at each survey for microhematuria. Results were similar to other models (Supplementary Table 2).

**Table 1.** Odds Ratios and 95% Credible Intervals From Bayesian Logistic Regression Models Comparing Morbidity-Positive Proportions Between Heavy-Intensity Prevalence Categories Within Surveys for *S. haematobium*–Related Morbidities

Morbidity	Survey	<1% vs ≥5%	1–5% vs ≥5%	<1% vs 1–5%
Any urinary bladder lesions	Baseline	**0.27 (0.21–0.34)**	**0.43 (0.33–0.54)**	**0.63 (0.47–0.85)**
	Follow-up 1	**0.22 (0.18–0.28)**	**0.52 (0.42–0.63)**	**0.43 (0.34–0.55)**
	Follow-up 2	**0.30 (0.22–0.40)**	**0.31 (0.22–0.43)**	0.98 (0.67–1.44)
Any upper urinary tract lesions	Baseline	0.90 (0.56–1.45)	0.77 (0.47–1.22)	1.18 (0.67–2.07)
	Follow-up 1	0.67 (0.41–1.10)	0.83 (0.51–1.34)	0.81 (0.46–1.42)
	Follow-up 2	0.61 (0.25–1.36)	0.36 (0.09–1.07)	1.68 (0.47–7.52)
Microhematuria	Baseline	**0.12 (0.10–0.15)**	**0.27 (0.23–0.31)**	**0.45 (0.37–0.56)**
	Follow-up 1	**0.14 (0.11–0.16)**	**0.77 (0.65–0.92)**	**0.18 (0.15–0.21)**
	Follow-up 2	**0.22 (0.18–0.28)**	**0.40 (0.31–0.51)**	**0.56 (0.43–0.73)**
Pain while urinating	Baseline	**0.62 (0.52–0.73)**	**0.67 (0.58–0.78)**	0.92 (0.77–1.11)
	Follow-up 1	0.95 (0.80–1.13)	1.28 (1.06–1.55)	**0.74 (0.62–0.89)**
	Follow-up 2	**0.69 (0.56–0.85)**	**0.70 (0.55–0.90)**	0.99 (0.77–1.27)

**Figure 2.**
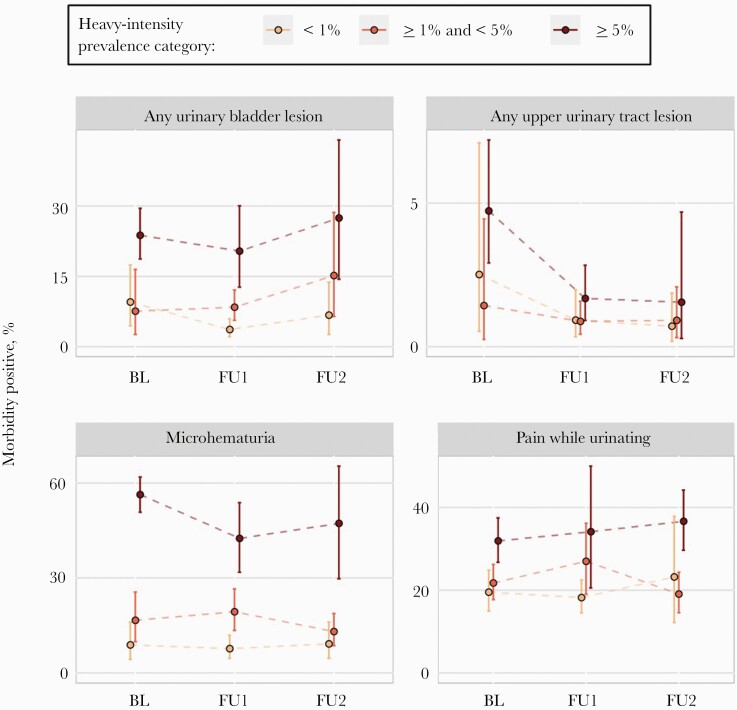
Line graphs of *Schistosoma haematobium*–related morbidity positive prevalence. Heavy-intensity prevalence category was determined at the school level. Participants were enrolled between 2003 and 2008 at each survey year (BL = baseline, FU1 = follow-up 1, FU2 = follow-up 2). Clustering by school was accounted for in 95% confidence bands. Infections were assessed by urine filtration. Model-based tests comparisons are included in Table 1. Plots of individual indicators and model-based comparisons are included in Supplementary Figure 3 and Supplementary Table 2, respectively.

Bold font indicates that the 95% Bayesian credible interval does not contain 1. Participants are school-age children, aged 5–15 years, enrolled between 2003 and 2008. Plots of unmodeled estimates are included in Figure 2.

Participants in schools with PHI <1% had lower odds of microhematuria as compared with participants in 1–5% PHI schools in all 3 surveys. For other measures, whether aggregated (Figure 2, Table 1) or individual (Supplementary Figure 2, Supplementary Table 2), the differences between morbidity in participants in schools with PHI <1% and participants in schools with 1–5% PHI was inconsistent, and for some urinary bladder indicators, morbidity was greater in participants in schools with PHI <1%.

The raw results (Figure 2; Supplementary Figure 2) differed at times from the modeled results, likely due to between-country variability. For example, the prevalence of any urinary bladder lesion varied at baseline between countries (Supplementary Figure 3). Regression models controlled for country-level differences, which account for the differences between the raw and modeled results.

### Schistosoma mansoni

For *S. mansoni*, the results from 5 to 65 schools with between 439 and 8890 participants per PHI category were analyzed (Supplementary Table 3). The overall prevalence of *S. mansoni* was lower than *S. haematobium*, and, as with *S. haematobium*, infection prevalence decreased in follow-up surveys, though this reduction was smaller than *S. haematobium* (Figure 1 A, right); the prevalence of most *S. mansoni* morbidities decreased during follow-up surveys (Figure 1 C; Supplementary Figure 4).

**Figure 3.**
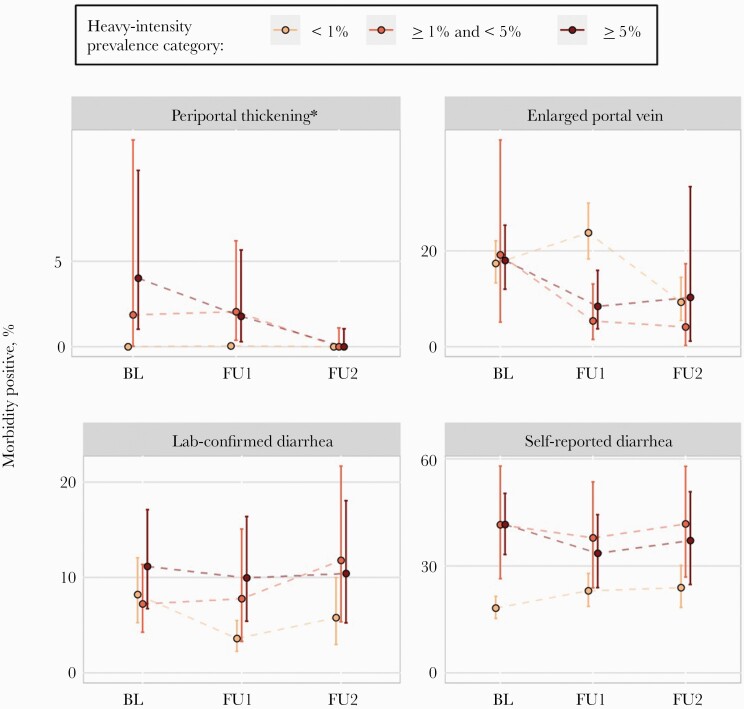
Line graphs of *Schistosoma mansoni*–related morbidity percentages. Heavy-intensity prevalence category was determined at the school level. Participants were enrolled between 2003 and 2008 at each survey year (BL = baseline, FU1 = follow-up 1, FU2 = follow-up 2). Clustering by school was accounted for in 95% confidence bands. Infections were assessed by Kato-Katz thick smears. Model-based test comparisons are included in Table 2. Plots of additional indicators and model-based comparisons are included in Supplementary Figure 4 and Supplementary Table 4, respectively.

As observed for *S. haematobium*, participants in schools with PHI <1% had lower odds of *S. mansoni*–associated morbidities than participants in schools with PHI ≥5% (Figures 3, Table 2; Supplementary Figure 5). At each survey, the odds were lower for participants in schools with PHI <1% compared with participants in schools with PHI ≥5% for laboratory-confirmed diarrhea. Periportal thickening (as measured by irregular image patterns C, D, E, or F) was lower at baseline, but not at follow-up 1. There was more self-reported diarrhea in participants in schools with PHI ≥5% compared with participants in schools with PHI <1% at baseline. In the unmodeled data, a greater proportion of children had an enlarged portal vein at the first follow-up in schools with PHI <1%, though this was largely driven by between-country variability, especially the much higher prevalence in Niger (Supplementary Figure 6). As country-level differences are accounted for in the modeling, the estimated odds of having an enlarged portal vein are lower in participants in <1% PHI schools than participants in schools with PHI ≥5% (baseline: OR, 0.41; 95% BCI, 0.32–0.52; follow-up 1: OR, 0.61; 95% BCI, 0.45–0.83; follow-up 2: OR, 0.57; 95% BCI, 0.38–0.86). However, comparisons between participants in schools with 1–5% PHI and participants in schools with PHI ≥5% produced inconsistent conclusions across surveys. For example, as expected, participants in schools with 1–5% PHI were at lower risk of having lab-confirmed diarrhea than participants in schools with PHI ≥5% at baseline (OR, 0.42; 95% BCI, 0.33–0.54) and follow-up 1 (OR, 0.63; 95% BCI, 0.47–0.85). But at follow-up 2, the relationship was reversed (OR, 1.37; 95% BCI, 0.97–1.94).

**Table 2.** Odds Ratios and 95% CIs From Bayesian Logistic Regression Models Comparing Morbidity-Positive Proportions Between Heavy-Intensity Prevalence Categories Within Surveys for *S. mansoni*–Related Morbidities

Morbidity	Survey	<1% vs ≥5%	1–5% vs ≥5%	<1% vs 1–5%
Image patterns C–F	Baseline	**0.02 (0.00–0.28)**	0.79 (0.29–2.11)	**0.03 (0.00–0.37)**
	Follow-up 1	0.20 (0.01–1.79)	1.80 (0.72–4.55)	0.11 (0.01–1.06)
	Follow-up 2		Nonestimable^a^	
Enlarged portal vein	Baseline	**0.41 (0.32–0.52)**	**0.59 (0.42–0.82)**	**0.70 (0.51–0.96)**
	Follow-up 1	**0.61 (0.45–0.83)**	0.82 (0.51–1.28)	0.75 (0.48–1.19)
	Follow-up 2	**0.57 (0.38–0.86)**	**0.51 (0.27–0.94)**	1.10 (0.66–1.94)
Laboratory-confirmed diarrhea	Baseline	**0.43 (0.35–0.54)**	**0.42 (0.33–0.54)**	1.01 (0.81–1.28)
	Follow-up 1	**0.38 (0.30–0.50)**	**0.63 (0.47–0.85)**	**0.61 (0.46–0.80)**
	Follow-up 2	**0.63 (0.45–0.87)**	1.37 (0.97–1.94)	**0.46 (0.34–0.62)**
Self-reported diarrhea	Baseline	**0.73 (0.65–0.82)**	1.08 (0.95–1.23)	**0.68 (0.60–0.77)**
	Follow-up 1	0.87 (0.75–1.00)	**0.71 (0.60–0.85)**	**1.22 (1.04–1.43)**
	Follow-up 2	0.93 (0.78–1.11)	1.01 (0.81–1.25)	0.92 (0.78–1.10)

Bold font indicates that the 95% credible interval does not contain 1. Participants are school-aged children, aged 5–15 years, enrolled between 2003 and 2008. Plots of unmodeled estimates are included in Figure 3.

^a^The odds ratios for image patterns C–F at follow-up 2 were highly variable due to the prevalence being close to 0. We chose to omit this effect due to its instability.

Findings were also inconsistent when comparing participants in schools with PHI <1% with participants in schools with 1–5% PHI. Participants in schools with PHI <1% compared with participants in schools with 1–5% PHI had lower odds of having an enlarged portal vein at baseline (OR, 0.70; 95% BCI, 0.51–0.96) but had increased odds at follow-up 2, though with considerable variability in the point estimate (OR, 1.10; 95% BCI, 0.66–1.94). For laboratory-confirmed diarrhea, participants in schools with PHI <1% had similar odds at baseline compared with participants in schools with 1–5% PHI (OR, 1.01; 95% BCI, 0.81–1.28) but had lower odds otherwise (follow-up 1: OR, 0.61; 95% BCI, 0.46–0.80; follow-up 2: OR, 0.46; 95% BCI, 0.34–0.62).

## DISCUSSION

In general, children in schools with PHI ≥5% displayed higher morbidity than children in schools with PHI <1% and 1–5% PHI, though the extent differed by species and was generally nonlinear, with participants in schools with PHI ≥5% having considerably higher rates. For *S. haematobium*, participants in schools with PHI <1% or 1–5% PHI had consistently lower odds of morbidity compared with participants in schools with PHI ≥5%. Thus, a targeted area with a prevalence of <5% *S. haematobium* heavy-intensity infections among school-age children is expected to have less morbidity than a targeted area with >5% heavy-intensity infections. For *S. mansoni*, while participants in schools with PHI <1% consistently had decreased odds of morbidity compared with participants in PHI ≥5% schools, this was not true for participants in schools with 1–5% PHI compared with participants in schools with PHI ≥5%. Participants in schools with 1–5% PHI sometimes had decreased odds of morbidity compared with participants in schools with PHI ≥5% and sometimes had increased odds. Therefore, for the *S. mansoni* morbidities that we measured, only the category of participants in schools with PHI <1% had lower morbidity than participants in schools with PHI ≥5%.

Microhematuria was the only morbidity for which there was a consistent difference between participants in schools with PHI 1–5% vs participants in schools with PHI <1%. These analyses underscore the correlation of microhematuria and *S. haematobium* infection. The WHO 2021–2030 neglected tropical disease roadmap [11] suggests defining an indicator for measuring morbidity of schistosomiasis as a critical action for achieving program goals within the next decade. Microhematuria, which has been used as a proxy for community-level *S. haematobium* prevalence for ~40 years [34] due to the 2 measures being correlated [35], would appear to be an ideal candidate for that species-specific indicator because reducing microhematuria to a prevalence consistent with non-schistosomiasis causes might indicate that urogenital schistosomiasis has been eliminated as a public health problem.

Other differences in morbidity between participants in schools with 1–5% PHI and PHI <1% were rare and often inconsistent. Occasional ORs greater or less than 1 were detected, but these associations were not realized at all surveys. There were also instances where participants in schools with PHI <1% had increased odds of morbidity compared with participants in schools with 1–5% PHI, such as the baseline presence of any upper urinary tract lesions for *S. haematobium* and follow-up 2 for periportal thickening for *S. mansoni*. Clearly, the PHI thresholds of 1% and 5% do not appear to correlate well with different levels of schistosomiasis morbidity across the 2 species for multiple morbidity indicators.

These analyses have some limitations and shortcomings. First, a single urine or stool sample was used in multiple countries, meaning the potential for diagnostic misclassification was higher than if more samples had been taken [36]. This is especially true for *S. mansoni* as the overall prevalence was lower, meaning a small number of missed heavy infections would have a greater impact on the PHI. This could explain some of the confusing results when comparing participants in schools with different PHI categories. In addition, our results are limited to morbidity in children. For some ultrasound-detectable morbidities, especially liver fibrosis for *S. mansoni,* substantial pathology does not appear until individuals have been infected for at least 10–15 years. More work is needed to better understand morbidity control in adults, especially as recent modeling studies have found utility in collecting adult data for both species [37, 38]. Reflecting the challenges associated with collecting field-based ultrasound data, many *S. mansoni* ultrasound components were not collected. A more complete ultrasound picture, especially including hepatomegaly and splenomegaly, would have allowed us to evaluate indicators of *S. mansoni* morbidity that are more common in children. Data for other schistosomiasis-associated morbidities often described for children, such as anemia, stunting, wasting, exercise intolerance, and delay in cognitive development, were not consistently collected by these control programs. If we had access to reliable data for these measures, we may have detected a relationship between PHI categories and morbidity in children. Another limitation was that for *S. mansoni*, most PHI ≥5% schools were in Uganda, potentially causing bias for comparisons involving the PHI ≥5% category. Although we cannot formally assess the bias, there is the potential that, if the prevalence of morbidity indicators among children in these PHI ≥5% Ugandan schools is higher than for children in other PHI ≥5% schools, differences between participants in PHI ≥5% schools and those in PHI <5% schools would be larger than they should be.

Our findings suggest that the global effort to control and eliminate schistosomiasis would benefit from revisiting how these goals are defined and operationalized. Given the lack of precision of current morbidity tools, especially in the context of low schistosomiasis prevalence, changes are needed for monitoring and evaluating schistosomiasis. Having 2 programmatic thresholds for schistosomiasis does not appear to have an empirical basis, and a single elimination as a public health problem target for determining when to stop mass annual preventive chemotherapy, such as is done with trachoma [39], would better suit schistosomiasis control programs. The recommended public health actions for countries that have controlled morbidity (1–5% PHI) are similar to countries that have not controlled morbidity (≥5% PHI) [12]. Only when a country is eligible for elimination as a public health problem (PHI <1%) do the public health actions change, which suggests that the control of morbidity category (1–5% PHI) is redundant. This is reflected in the current road map, which only mentions PHI <1% [11]. In addition, most programs have initiated treatment or are currently below 5% PHI [40]. Still, it is somewhat disappointing that PHI <1% and 1–5% PHI rarely show useful significant differences.

A different measure is needed for morbidity control. Most programs only collect prevalence data for decision-making on frequency of preventive chemotherapy and do not calculate PHI. PHI is rarely utilized in practice, and as these results demonstrate a lack of association between PHI and morbidity indicator levels, morbidity categorization by PHI may be superfluous. Because morbidity may be experienced by people with all infection intensities [2, 16], utilizing prevalence of any infection may better predict the morbidity status of a school or community.

Any new measure for morbidity control or elimination as a public health problem needs to have evidence-based targets that better align with actual morbidity [17], and hopefully new targets can be developed. Development of guidelines for elimination of schistosomiasis as a public health intervention needs deeper thought into which morbidity indicators should be used and operationalized. There is a wide array of possible indicators, and it is unclear whether pegging program targets to a single indicator is optimal or whether an aggregate measure would provide greater utility. Chosen indicators should be tailored toward the specific age groups sampled and be clinically meaningful, readily measured (preferably in the field), easily interpreted by control programs, and prevalent in the age groups that are sampled. A limiting factor in ultrasonic indicators is that they are difficult to collect in the field, but advances in technology are making ultrasound evaluations more practical [41] and may mean that these indicators can be used to assess morbidity control.

Finally, even though microhematuria appears to be a good candidate for monitoring *S. haematobium* morbidity in school-age children, it is the sole example. This demonstrates the need for a comprehensive exploration of the associations between community-level infection and morbidity after initiation of MDA. Such explorations of measuring morbidity should be undertaken, with an emphasis on estimating the background levels of morbidity and quantifying the relationship between school and community infection and morbidity levels. One such pilot initiative has begun in an *S. haematobium–*endemic area and an *S. mansoni*–endemic area [42], but hopefully identical or similar study designs can be performed in different countries and ecologic archetypes, especially foci with mixed *S. haematobium* and *S. mansoni* infections and *S. japonicum*–endemic areas, as well as different age groups. Finally, while some information exists on how an infected child’s ultrasound evaluation changes over time [43], greater knowledge of people’s ultrasound indicator progression postinfection is needed, especially in older age groups.

## CONCLUSIONS

The success of schistosomiasis programs is tied to the WHO categorizations for morbidity control. Those categories are based on PHI, which our analyses demonstrate often have similar and overlapping levels of morbidity. Thus, the usefulness of PHI in defining control status is limited, which indicates that, for the goal of elimination of this public health problem as outlined in the WHO 2021–2030 neglected tropical disease roadmap [11] to be met, community measurements must be better aligned with schistosomiasis-related morbidity levels. A reconfiguration of these morbidity categories is warranted.

## Supplementary Data

Supplementary materials are available at *Open Forum Infectious Diseases* online. Consisting of data provided by the authors to benefit the reader, the posted materials are not copyedited and are the sole responsibility of the authors, so questions or comments should be addressed to the corresponding author.

ofab179_suppl_Supplementary_Materials
