# Supplementary material for: Control and Elimination of Schistosomiasis as a Public Health Problem: Thresholds Fail to Differentiate Schistosomiasis Morbidity Prevalence in Children
Source: Open Forum Infect Dis. 2021 Apr 15;8(7):ofab179. doi: 10.1093/ofid/ofab179 (PMC8297701; doi:10.1093/ofid/ofab179)
Supplement: ofab179_suppl_Supplementary_Materials [file ofab179_suppl_supplementary_materials.docx]

Supplemental materials for “Control and elimination of schistosomiasis as a public health problem: thresholds fail to differentiate schistosomiasis morbidity prevalence in children”.

Ryan E. Wiegand^1,2,3*^, W. Evan Secor^1^, Fiona M. Fleming^4^, Michael D. French^5^, Charles H. King^6^_,_ Susan P. Montgomery^1^, Darin Evans^7^, Jürg Utzinger^2,3^, Penelope Vounatsou^2,3^, Sake J. de Vlas^8^

1. Division of Parasitic Diseases and Malaria, Centers for Disease Control and Prevention, Atlanta, GA, United States of America
2. Swiss Tropical and Public Health Institute, Basel, Switzerland
3. University of Basel, Basel, Switzerland
4. Schistosomiasis Control Initiative, London, United Kingdom
5. RTI International, Washington DC, United States of America
6. Center for Global Health and Diseases, Case Western Reserve University, Cleveland, Ohio, United States of America
7. United States Agency for International Development, Washington, DC, United States of America
8. Department of Public Health, Erasmus MC, University Medical Center Rotterdam, Rotterdam, The Netherlands

Contents

[Statistical Methods 4](#_Toc71948395)

[Details 4](#_Toc71948396)

[Implementation 5](#_Toc71948397)

[JAGS model code for logistic model 6](#_Toc71948398)

[JAGS model code for binomial model 7](#_Toc71948399)

[Supplemental Figures 9](#_Toc71948400)

[Supplementary Figure 1. Line graphs of the percentage of 6-15 year-old children who were *Schistosoma haematobium*-related morbidity positive at each survey year (BL=baseline, FU1=follow up 1, FU2=follow up 2) not included in Figure 1, row B. Clustering by school accounted for in 95% confidence bands. 9](#_Toc71948401)

[Supplementary Figure 2. Line graphs of *Schistosoma haematobium*-related morbidity percentages not included in Figure 1. Heavy-intensity prevalence category determined at the school-level. Participants were enrolled between 2003 and 2008 at each survey year (BL=baseline, FU1=follow up 1, FU2=follow up 2). Clustering by school accounted for in 95% confidence bands. Infections were assessed by urine filtration. Model-based tests comparisons are included in Supplementary Table 2. Plots of aggregated indicators, microhematuria, and self-reported pain while urinating are included in Figure 2. 10](#_Toc71948402)

[Supplementary Figure 3. Line graphs of any urinary bladder lesion percentage by heavy intensity prevalence category by country across three surveys (baseline, BL; follow-up 1, FU1; follow-up2, FU2). Participants were enrolled between 2003 and 2008. Clustering by school accounted for in 95% confidence bands. Infections were assessed by urine filtration. 11](#_Toc71948403)

[Supplementary Figure 4. Line graphs of the percentage of 6-15 year-old school children who were *Schistosoma mansoni*-related morbidity positive at each survey year (BL=baseline, FU1=follow up 1, FU2=follow up 2). Clustering by school accounted for in 95% confidence bands. Selected indicators here are included in Figure 1, row C. 12](#_Toc71948404)

[Supplementary Figure 5. Line graphs of *Schistosoma mansoni*-related morbidity percentages. Heavy-intensity prevalence category determined at the school-level. Participants were enrolled between 2003 and 2008 at each survey year (BL=baseline, FU1=follow up 1, FU2=follow up 2). Clustering by school accounted for in 95% confidence bands. Infections were assessed by Kato-Katz thick smears. Model-based tests comparisons are included in Supplementary Table 4. Plots of other indicators are included in Figure 3. 13](#_Toc71948405)

[Supplementary Figure 6. Line graphs of enlarged portal vein percentage by heavy intensity prevalence category by country across three surveys (baseline, BL; follow-up 1, FU1; follow-up2, FU2). Participants were enrolled between 2003 and 2008. Clustering by school accounted for in 95% confidence bands. Infections were assessed by Kato-Katz thick smears. 14](#_Toc71948406)

[Supplemental Tables 15](#_Toc71948407)

[Supplementary Table 1. Sample sizes of schools and participants in *S. haematobium* analyses*.* Frequencies are broken down by morbidity and heavy-intensity prevalence category. Cells contain the number of schools with the number of participants in parentheses. Participants are school-aged children, with morbidity and infection data, aged 6-15 years, enrolled between 2003 and 2008. 15](#_Toc71948408)

[Table S2. Odds ratios and 95% credible intervals from Bayesian logistic regression models comparing morbidity positive proportions between heavy-intensity prevalence categories within surveys for *S. haematobium*-related morbidities not presented in Table 1. Bold font indicates the 95% credible interval does not contain one. Participants are school-aged children, aged 6-15 years, and enrolled between 2003 and 2008. Corresponding plots of unmodeled morbidity prevalence are in Figure 2 and Supplementary Figure 3. 17](#_Toc71948409)

[Supplementary Table 3. Sample sizes of schools and participants in *S. mansoni* analyses. Frequencies are broken down by morbidity and heavy-intensity prevalence category. Cells contain the number of schools with the number of participants in parentheses. Participants are school-age children, with morbidity and infection data, aged 6-15 years, and enrolled between 2003 and 2008. 18](#_Toc71948410)

[Supplementary Table 4. Odds ratios and 95% credible intervals from Bayesian logistic regression models comparing morbidity positive proportions between heavy-intensity prevalence categories within surveys for S. mansoni-related morbidities not presented in Table 2. Bold font indicates the 95% credible interval does not contain one. Participants are school-age children, aged 5-15 years, and enrolled between 2003 and 2008. Corresponding plots of unmodeled morbidity prevalence are in Figure 3 and Supplementary Figure 4. 19](#_Toc71948411)

[Bibliography 20](#_Toc71948412)

# Statistical Methods

## Details

Our outcome in all models was the presence of a morbidity, $y_{i}$, where

$y_{i}=\left\{ \begin{aligned} 0, \text{if negative} \\ 1, \text{if positive} \end{aligned} \right.$.

We assume

$$y_{i}\sim\text{Bernoulli}(p_{i})$$

where $i$ denotes an observation. $p_{i}$ is forced to be bounded on $\left[ 0,1 \right]$. For the logistic model, we assume a logit transform of the linear predictor, specifically

$$p_{i}\text{=}\frac{e^{\eta_{i}}}{{1+e}^{\eta_{i}}}$$

where $\eta_{i}$is the linear predictor. The form of $\eta_{i}$ depends on whether participant was sampled in multiple waves. We had $n$ observations in the dataset, where ${i=1,...,n}_{1}$ come from children who were only sampled once during these three surveys and ${i=n_{1}+1,..., n}_{2}$ come from children who were only sampled more than once. For ${i=1,...,n}_{1}$ ,

$$\eta_{i}= \beta_{0}+\beta_{1}*\text{COM}_{i}+\beta_{2}*\text{EPHP}_{i}+ \beta_{3}*\text{FU1}_{i}+\beta_{4}*\text{FU2}_{i} +\beta_{5}*\text{COM}_{i}*\text{FU1}_{i}+\beta_{6}*\text{COM}_{i}*\text{FU2}_{i}+ \beta_{7}*\text{EPHP}_{i}*\text{FU1}_{i}+\beta_{8}*\text{EPHP}_{i}*\text{FU2}_{i}+\beta_{9_{1}}*\text{age}_{1,i}+\cdots+ \beta_{9_{j}}*\text{age}_{j,i}+\beta_{10}*\text{female}_{i}+\beta_{{11}_{1}}*\text{country}_{1,i}+\cdots+ \beta_{{11}_{j}}*\text{country}_{j,i}+\gamma_{1,k}$$

and for ${i=n_{1}+1,..., n}_{2}$ ,

$$\eta_{i}= \beta_{0}+\beta_{1}*\text{COM}_{i}+\beta_{2}*\text{EPHP}_{i}+ \beta_{3}*\text{FU1}_{i}+\beta_{4}*\text{FU2}_{i} +\beta_{5}*\text{COM}_{i}*\text{FU1}_{i}+\beta_{6}*\text{COM}_{i}*\text{FU2}_{i}+ \beta_{7}*\text{EPHP}_{i}*\text{FU1}_{i}+\beta_{8}*\text{EPHP}_{i}*\text{FU2}_{i}+\beta_{9_{1}}*\text{age}_{1,i}+\cdots+ \beta_{9_{j}}*\text{age}_{j,i}+\beta_{10}*\text{female}_{i}+\beta_{{11}_{1}}*\text{country}_{1,i}+\cdots+ \beta_{{11}_{j}}*\text{country}_{j,i}+\gamma_{1,k}+\gamma_{2,l}$$

In these equations,

- $\beta$’s are the coefficient estimates (on the log odds scale),
- $\text{COM}_{i}$ is an indicator variable denoting whether observation $i$ is from a school that has PHI≤5% and PHI>1%,
- $\text{EPHP}_{i}$is an indicator variable denoting whether observation $i$ is from a school that has PHI≤1%,
- $\text{FU1}_{i}$equals 1 when observation $i$ was ascertained in follow up 1,
- $\text{FU2}_{i}$equals 1 when observation $i$ was ascertained in follow up 2,
- $\text{age}_{i}$ is an indicator variable for the age of the participant treated as a category with 6 as the reference category to ages 7 to 15,
- $\text{female}_{i}$ is an indicator variable for whether participant is a female with male as the reference category,
- $\text{country}_{i}$ is an indicator variable for the country of the observation with $j$ countries in each model where $j$ depends on the morbidity studied, and
- $\gamma_{1,k}$ and $\gamma_{2,l}$ are random effects for school and person, respectively, where an observation is from school $k$ and person $l$, with the latter only for ${i=n_{1}+1,..., n}_{2}$.

All $\beta$’s have a Cauchy prior distribution with a center of zero and a scale of 2.5.[1] For the random effect for school, $\gamma_{1,k}\sim\text{Normal}\left( 0,\tau_{1} \right)$ where $\tau_{1}={\rho_{1}}^{-2}$ and $\rho_{1}$ is given a scaled Gamma prior with 1 degree of freedom and a scale of 25. This is equivalent to the standard deviation being distributed as a half-t distribution.[2] The random effect for person follows similarly.

Binomial models are fit in the same way, except $y_{i}\sim Bn(t_{i},p_{i})$ where $y_{i}$ is the number of morbidity indicators present and $t_{i}$is the total number of morbidity indicator tests completed for observation $i$.

Specific estimates for the three PHI categories (PHI < 1%; PHI ≥ 1% and < 5%; and PHI ≥ 5%) by survey are then estimated by contrast statements.

## Implementation

Models were fit via Markov Chain Monte Carlo using JAGS[3] and CODA[4] in R via the rjags package.[5] Three chains were fit with an adaptive phase of 20,000 iterations per chain. For the final model, the iterations from the adaptive phase were discarded and each chain was run for another 100,000 iterations. After completing the 100,000 iterations, graphical displays of the trace and densities functions were used to determine if any chains or a subset of iterations should be discarded. Results were then summarized.

## JAGS model code for logistic model

model {

for (i in 1:n.single) {

y.1[i] ~ dbin(p.bound.1[i], 1)

p.bound.1[i] <- max(0, min(1, p.1[i]))

logit(p.1[i]) <- eta.1[i]

eta.1[i] <- inprod(b[1:P], X.1[i,1:P]) + gamma1[school[i]]

}

for (i in 1:n.multi) {

y.2[i] ~ dbin(p.bound.2[i], 1)

p.bound.2[i] <- max(0, min(1, p.2[i]))

logit(p.2[i]) <- eta.2[i]

eta.2[i] <- inprod(b[1:P], X.2[i,1:P]) + gamma1[school[i]] + gamma2[multi.person[i]]

}

# setting up random effect 1

for (j in 1:n.school) {

gamma1[j] ~ dnorm(0, tau1)

}

tau1 <- pow(rho1, -2)

rho1 ~ dscaled.gamma(25, 1)

# setting up random effect 2

for (j in 1:n.multi.person) {

gamma2[j] ~ dnorm(0, tau2)

}

tau2 <- pow(rho2, -2)

rho2 ~ dscaled.gamma(25, 1)

# priors for fixed effects

for (f in 1:P) {

b[f] ~ dt(0, pow(2.5,-2), 1)

}

# contrasts

c[1] <- b[4]

c[2] <- b[4] + b[6]

c[3] <- b[4] + b[7]

c[4] <- b[5]

c[5] <- b[5] + b[8]

c[6] <- b[5] + b[9]

c[7] <- b[5] - b[4]

c[8] <- b[5] + b[8] - b[4] - b[6]

c[9] <- b[5] + b[9] - b[4] - b[7]

c[10] <- b[2]

c[11] <- b[3]

c[12] <- b[3] - b[2]

c[13] <- b[2] + b[6]

c[14] <- b[3] + b[7]

c[15] <- b[3] + b[7] - b[2] - b[6]

c[16] <- b[2] + b[8]

c[17] <- b[3] + b[9]

c[18] <- b[3] + b[9] - b[2] - b[8]

# exceedence probabilities

for (g in 1:P) { pr.b[g] <- step(b[g]) }

for (h in 1:18) { pr.c[h] <- step(c[h]) }

}

## JAGS model code for binomial model

model {

for (i in 1:n.1) {

y.1[i] ~ dbin(p.1[i], n.tests.1[i])

logit(p.1[i]) <- eta.1[i]

model {

for (i in 1:n.single) {

y.1[i] ~ dbin(p.1[i], t.1[i])

logit(p.1[i]) <- eta.1[i]

eta.1[i] <- inprod(b[1:P], X.1[i,1:P]) + gamma1[school[i]]

}

for (i in 1:n.multi) {

y.2[i] ~ dbin(p.2[i], t.2[i])

logit(p.2[i]) <- eta.2[i]

eta.2[i] <- inprod(b[1:P], X.2[i,1:P]) + gamma1[school[i]] + gamma2[multi.person[i]]

}

# setting up random effect 1

for (j in 1:n.school) {

gamma1[j] ~ dnorm(0, tau1)

}

tau1 <- pow(rho1, -2)

rho1 ~ dscaled.gamma(25, 1)

# setting up random effect 2

for (j in 1:n.multi.person) {

gamma2[j] ~ dnorm(0, tau2)

}

tau2 <- pow(rho2, -2)

rho2 ~ dscaled.gamma(25, 1)

# priors for fixed effects

for (f in 1:P) {

b[f] ~ dt(0, pow(2.5,-2), 1)

}

# contrasts

c[1] <- b[4]

c[2] <- b[4] + b[6]

c[3] <- b[4] + b[7]

c[4] <- b[5]

c[5] <- b[5] + b[8]

c[6] <- b[5] + b[9]

c[7] <- b[5] - b[4]

c[8] <- b[5] + b[8] - b[4] - b[6]

c[9] <- b[5] + b[9] - b[4] - b[7]

c[10] <- b[2]

c[11] <- b[3]

c[12] <- b[3] - b[2]

c[13] <- b[2] + b[6]

c[14] <- b[3] + b[7]

c[15] <- b[3] + b[7] - b[2] - b[6]

c[16] <- b[2] + b[8]

c[17] <- b[3] + b[9]

c[18] <- b[3] + b[9] - b[2] - b[8]

# exceedence probabilities

for (g in 1:P) { pr.b[g] <- step(b[g]) }

for (h in 1:18) { pr.c[h] <- step(c[h]) }

}

# Supplemental Figures

## Supplementary Figure 1. Line graphs of the percentage of 6-15 year-old children who were *Schistosoma haematobium*-related morbidity positive at each survey year (BL=baseline, FU1=follow up 1, FU2=follow up 2) not included in Figure 1, row B. Clustering by school accounted for in 95% confidence bands.


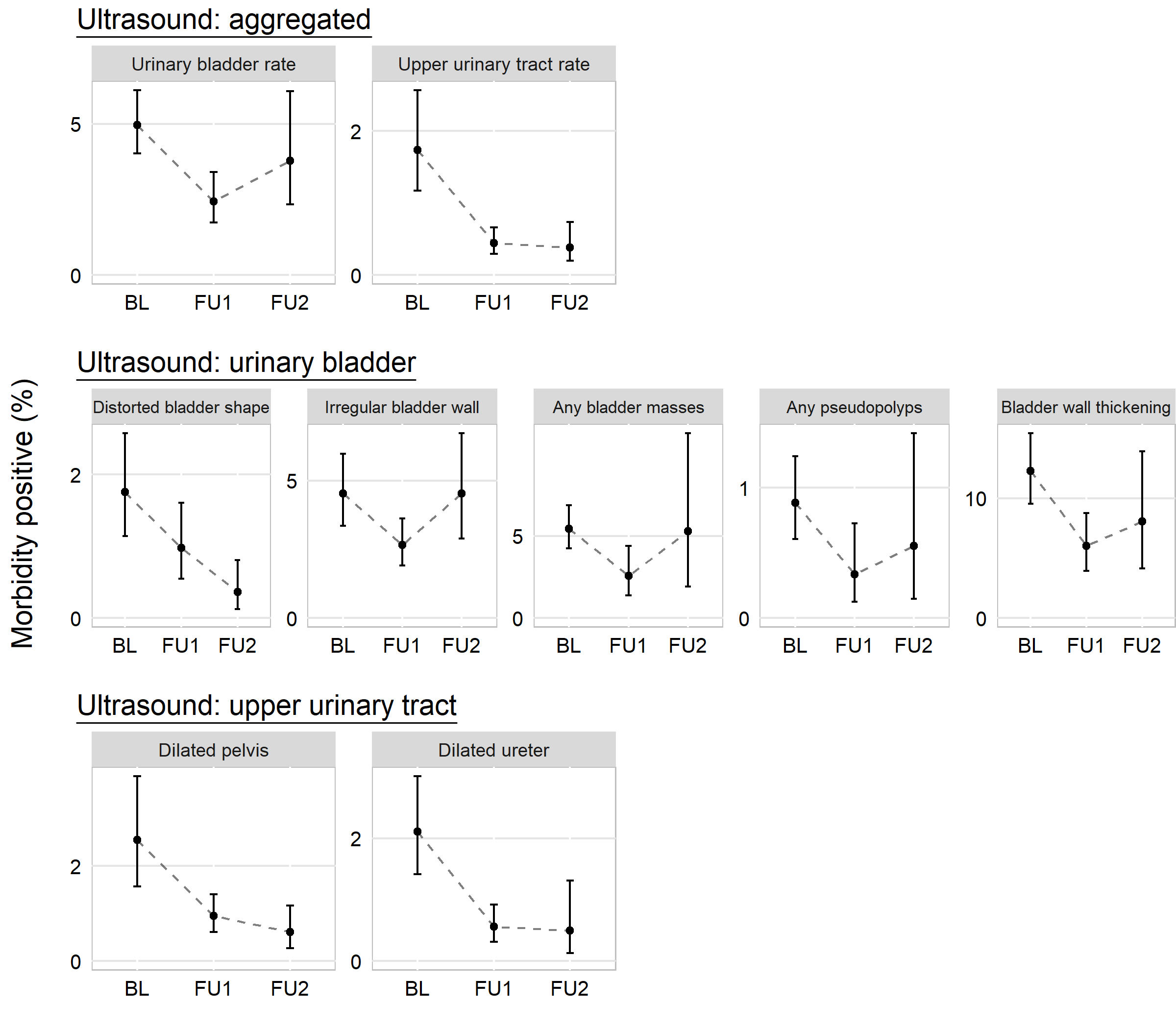


Note: Aggregated ultrasound measures include both dilated left and right pelvis and both dilated left and right ureters, but results shown for dilated pelvis and dilated ureter combine the left and right indicators into a single, binary variable of present in either or not present in either.

## Supplementary Figure 2. Line graphs of *Schistosoma haematobium*-related morbidity percentages not included in Figure 1. Heavy-intensity prevalence category determined at the school-level. Participants were enrolled between 2003 and 2008 at each survey year (BL=baseline, FU1=follow up 1, FU2=follow up 2). Clustering by school accounted for in 95% confidence bands. Infections were assessed by urine filtration. Model-based tests comparisons are included in Supplementary Table 2. Plots of aggregated indicators, microhematuria, and self-reported pain while urinating are included in Figure 2.


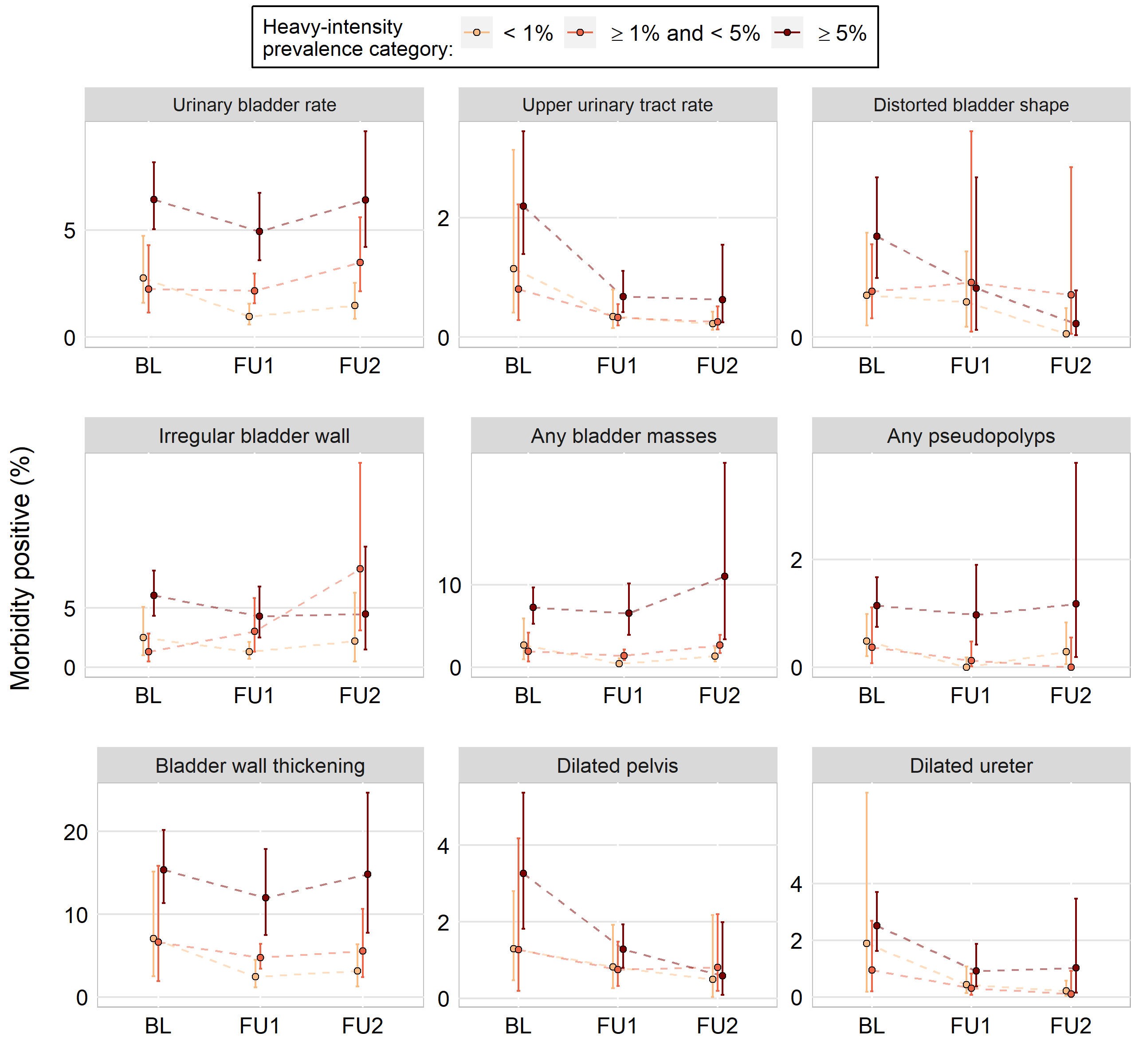


Note: Aggregated ultrasound measures include both dilated left and right pelvis and both dilated left and right ureters, but results shown for dilated pelvis and dilated ureter combine the left and right indicators into a single, binary variable of present in either or not present in either.

## Supplementary Figure 3. Line graphs of any urinary bladder lesion percentage by heavy intensity prevalence category by country across three surveys (baseline, BL; follow-up 1, FU1; follow-up2, FU2). Participants were enrolled between 2003 and 2008. Clustering by school accounted for in 95% confidence bands. Infections were assessed by urine filtration.


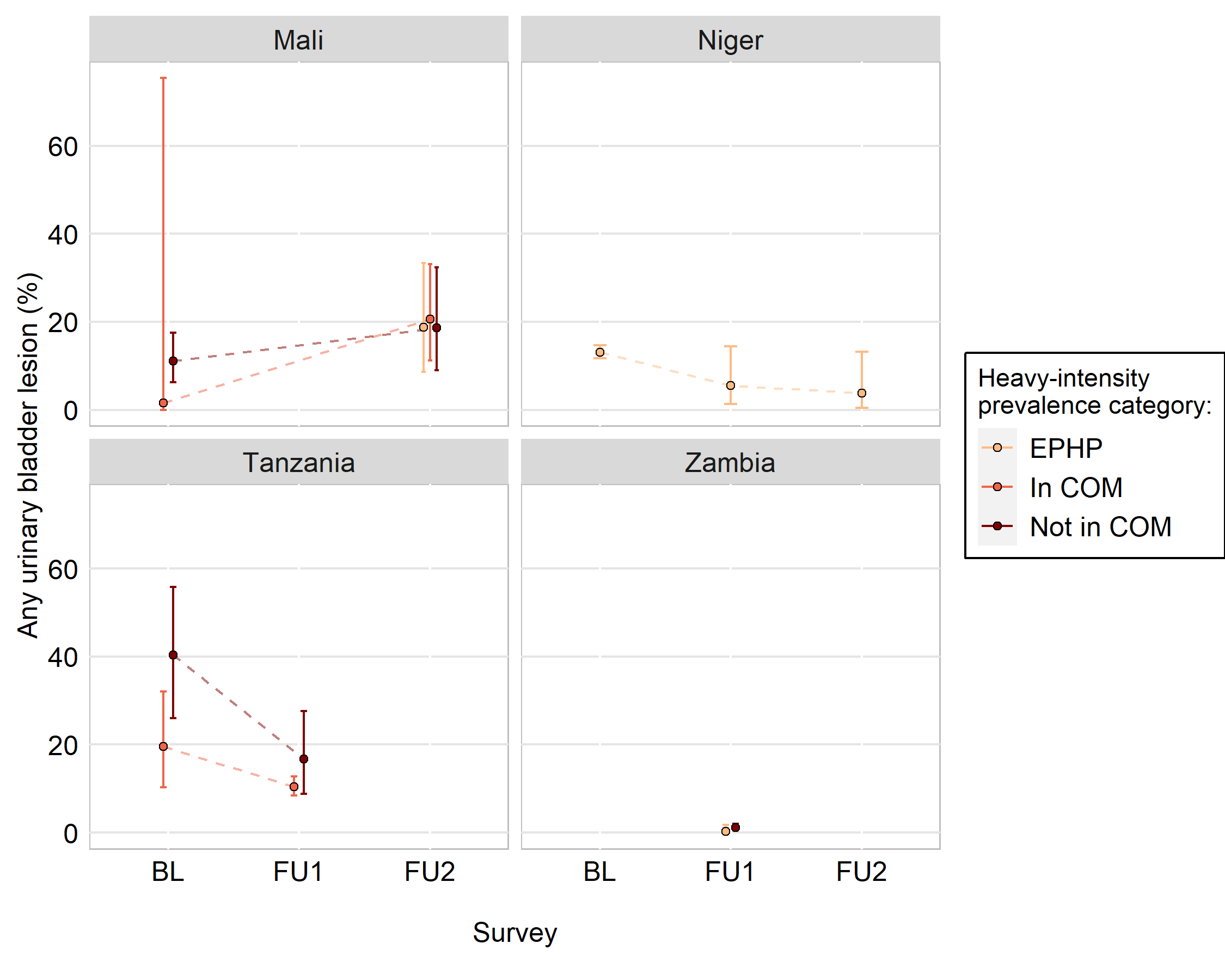


## Supplementary Figure 4. Line graphs of the percentage of 6-15 year-old school children who were *Schistosoma mansoni*-related morbidity positive at each survey year (BL=baseline, FU1=follow up 1, FU2=follow up 2). Clustering by school accounted for in 95% confidence bands. Selected indicators here are included in Figure 1, row C.


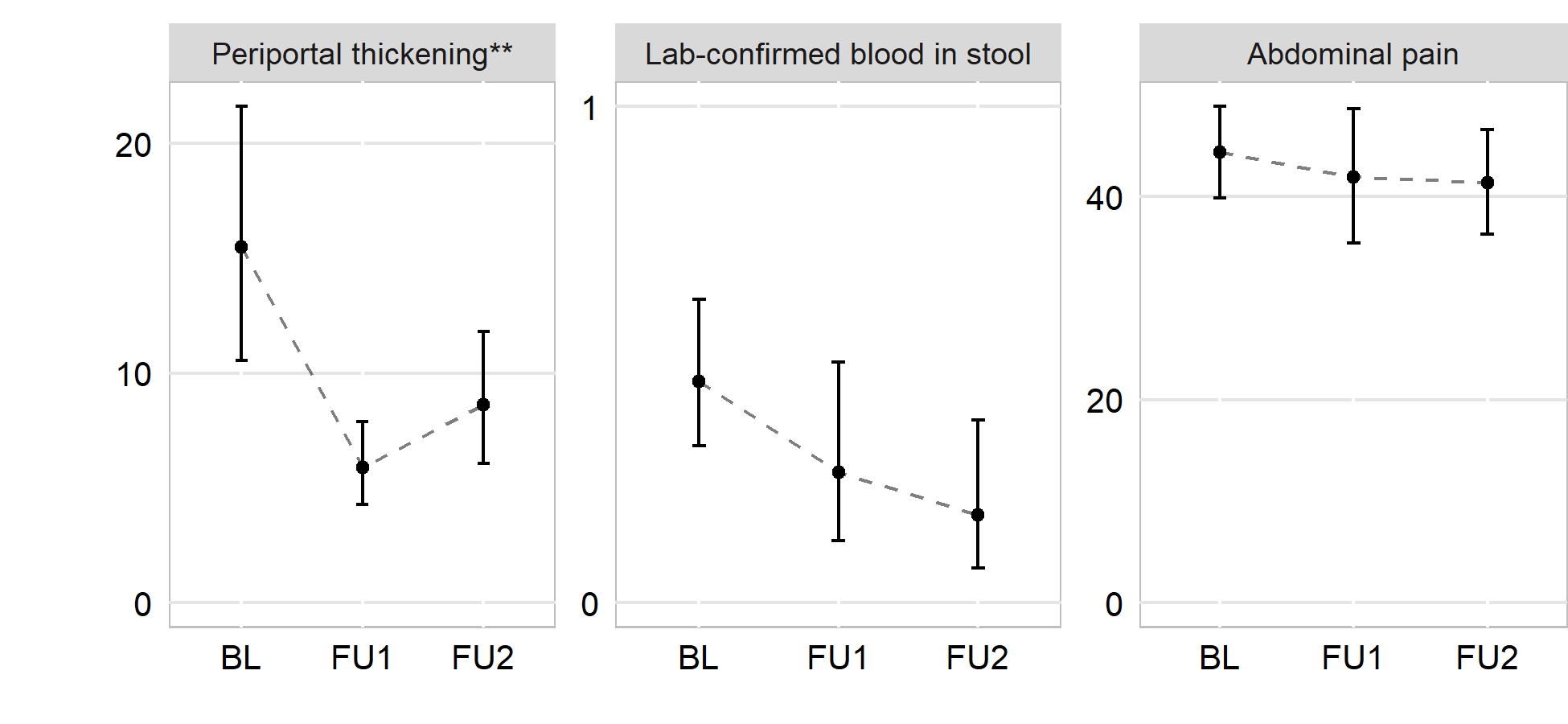


**Periportal thickening is measured by any one of image pattern B, C, D, E, or F.

## Supplementary Figure 5. Line graphs of *Schistosoma mansoni*-related morbidity percentages. Heavy-intensity prevalence category determined at the school-level. Participants were enrolled between 2003 and 2008 at each survey year (BL=baseline, FU1=follow up 1, FU2=follow up 2). Clustering by school accounted for in 95% confidence bands. Infections were assessed by Kato-Katz thick smears. Model-based tests comparisons are included in Supplementary Table 4. Plots of other indicators are included in Figure 3.


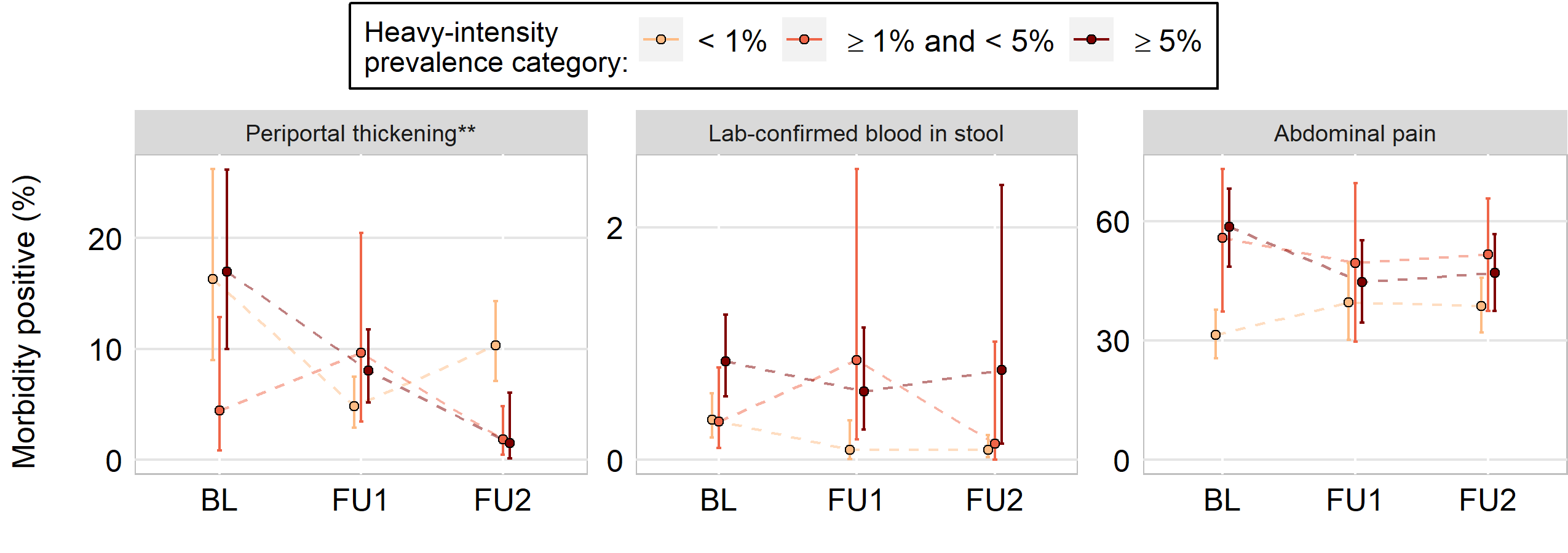


**Periportal thickening is measured by any one of image pattern B, C, D, E, or F.

## Supplementary Figure 6. Line graphs of enlarged portal vein percentage by heavy intensity prevalence category by country across three surveys (baseline, BL; follow-up 1, FU1; follow-up2, FU2). Participants were enrolled between 2003 and 2008. Clustering by school accounted for in 95% confidence bands. Infections were assessed by Kato-Katz thick smears.


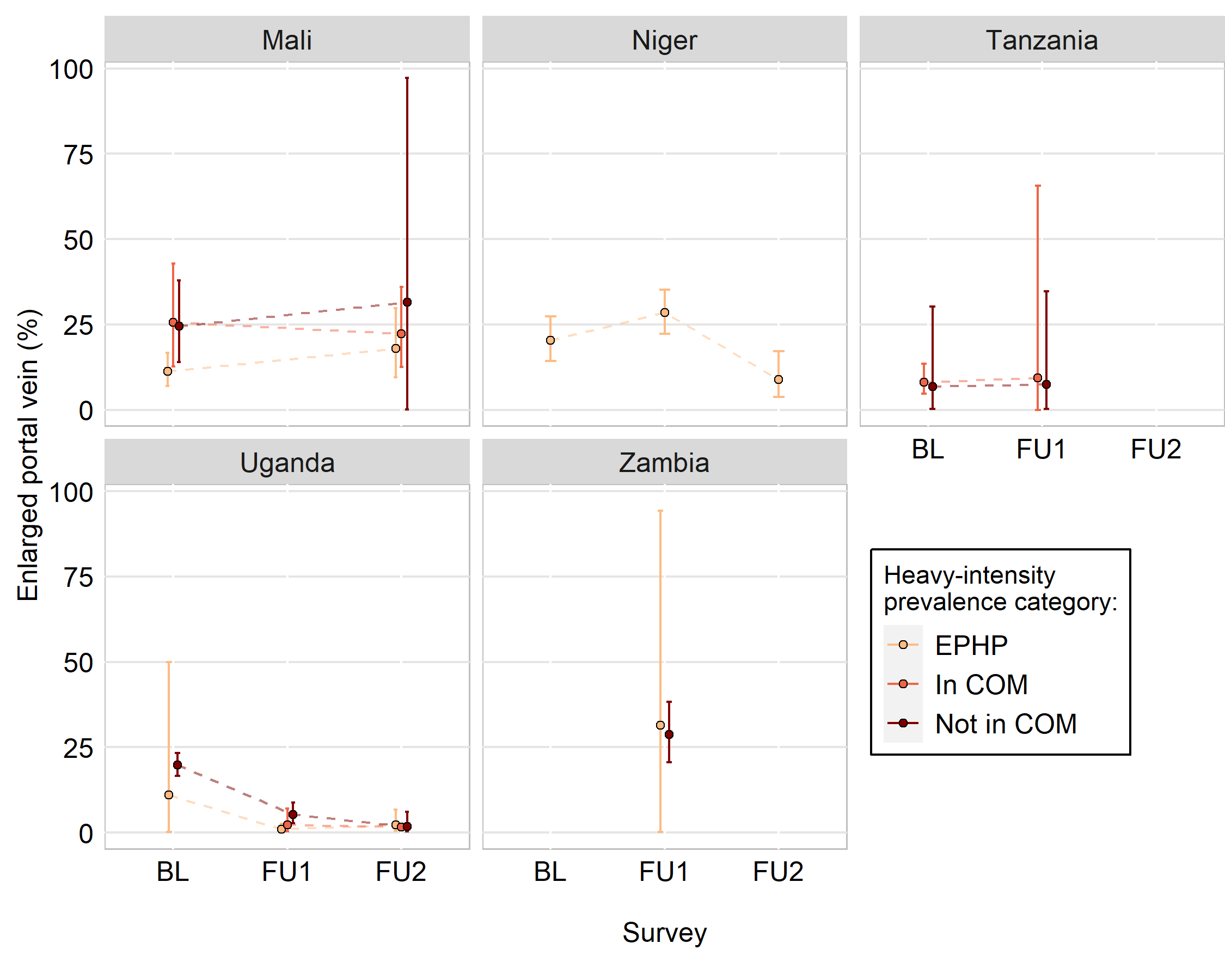


# Supplemental Tables

## Supplementary Table 1. Sample sizes of schools and participants in *S. haematobium* analyses*.* Frequencies are broken down by morbidity and heavy-intensity prevalence category. Cells contain the number of schools with the number of participants in parentheses. Participants are school-aged children, with morbidity and infection data, aged 6-15 years, enrolled between 2003 and 2008.

|  | | Schools (Participants) | | |
| --- | --- | --- | --- | --- |
| Morbidity | Survey | Heavy-intensity prevalence < 1% | Heavy-intensity prevalence ≥ 1% and < 5% | Heavy-intensity prevalence ≥ 5% |
|  |  |  |  |  |
| *Ultrasound: aggregated* |  |  |  |  |
| Any urinary bladder | Baseline | 12 (1635) | 12 (1887) | 44 (6186) |
| lesions | Follow up 1 | 16 (2704) | 9 (1586) | 11 (1957) |
|  | Follow up 2 | 7 (1395) | 8 (861) | 7 (1343) |
| Any upper urinary tract | Baseline | 12 (1628) | 12 (1882) | 44 (6172) |
| lesions | Follow up 1 | 16 (2692) | 9 (1589) | 11 (1957) |
|  | Follow up 2 | 7 (1398) | 8 (867) | 7 (1350) |
| Urinary bladder rate | Baseline | 12 (1752) | 12 (1909) | 44 (6275) |
|  | Follow up 1 | 16 (2814) | 9 (1660) | 11 (1980) |
|  | Follow up 2 | 7 (1420) | 8 (875) | 7 (1363) |
| Upper urinary tract rate | Baseline | 12 (1752) | 12 (1904) | 44 (6267) |
|  | Follow up 1 | 16 (2813) | 9 (1662) | 11 (1980) |
|  | Follow up 2 | 7 (1421) | 8 (876) | 7 (1363) |
|  |  |  |  |  |
| *Ultrasound: urinary bladder* | |  |  |  |
| Distorted bladder shape | Baseline | 16 (2707) | 9 (1586) | 11 (1957) |
|  | Follow up 1 | 7 (1400) | 8 (864) | 7 (1345) |
|  | Follow up 2 | 12 (1641) | 12 (1892) | 44 (6213) |
| Irregular bladder wall | Baseline | 16 (2708) | 9 (1592) | 11 (1957) |
|  | Follow up 1 | 7 (1400) | 8 (865) | 7 (1355) |
|  | Follow up 2 | 12 (1642) | 12 (1894) | 44 (6219) |
| Any bladder masses | Baseline | 16 (2707) | 9 (1592) | 11 (1957) |
|  | Follow up 1 | 7 (1399) | 8 (865) | 7 (1355) |
|  | Follow up 2 | 12 (1641) | 12 (1893) | 44 (6213) |
| Any pseudopolyps | Baseline | 16 (2706) | 9 (1592) | 11 (1957) |
|  | Follow up 1 | 7 (1400) | 8 (866) | 7 (1356) |
|  | Follow up 2 | 12 (1642) | 12 (1893) | 44 (6210) |
| Bladder wall thickening | Baseline | 16 (2708) | 9 (1592) | 11 (1957) |
|  | Follow up 1 | 7 (1400) | 8 (865) | 7 (1356) |
|  | Follow up 2 | 16 (2707) | 9 (1586) | 11 (1957) |
|  |  |  |  |  |
| *Ultrasound: upper urinary tract* | |  |  |  |
| Dilated left or right | Baseline | 12 (1629) | 12 (1886) | 44 (6187) |
| pelvis | Follow up 1 | 16 (2692) | 9 (1592) | 11 (1957) |
|  | Follow up 2 | 7 (1400) | 8 (867) | 7 (1353) |
| Dilated left or right | Baseline | 12 (1641) | 12 (1888) | 44 (6195) |
| Ureter | Follow up 1 | 16 (2705) | 9 (1591) | 11 (1957) |
|  | Follow up 2 | 7 (1400) | 8 (867) | 7 (1353) |
|  |  |  |  |  |
| *Laboratory and self-report* | |  |  |  |
| Microhematuria | Baseline | 15 (1963) | 14 (2147) | 59 (7766) |
|  | Follow up 1 | 19 (3221) | 10 (1725) | 11 (1969) |
|  | Follow up 2 | 7 (1409) | 8 (894) | 7 (1369) |
| Pain while urinating | Baseline | 15 (1953) | 14 (2130) | 49 (6918) |
|  | Follow up 1 | 19 (3208) | 10 (1719) | 11 (1961) |
|  | Follow up 2 | 7 (1403) | 8 (869) | 7 (1365) |

## Table S2. Odds ratios and 95% credible intervals from Bayesian logistic regression models comparing morbidity positive proportions between heavy-intensity prevalence categories within surveys for *S. haematobium*-related morbidities not presented in Table 1. Bold font indicates the 95% credible interval does not contain one. Participants are school-aged children, aged 6-15 years, and enrolled between 2003 and 2008. Corresponding plots of unmodeled morbidity prevalence are in Figure 2 and Supplementary Figure 3.

| Morbidity | Survey | < 1% vs. ≥ 5% | 1-5% v. ≥ 5% | < 1% v. 1-5% |
| --- | --- | --- | --- | --- |
|  |  |  |  |  |
| *Ultrasound: aggregated* |  |  |  |  |
| Urinary bladder rate | Baseline | **0.42 (0.35, 0.51)** | **0.65 (0.53, 0.79)** | **0.65 (0.52, 0.82)** |
|  | Follow up 1 | **0.43 (0.37, 0.51)** | **0.75 (0.64, 0.88)** | **0.58 (0.48, 0.69)** |
|  | Follow up 2 | **0.36 (0.28, 0.46)** | **0.41 (0.31, 0.53)** | 0.89 (0.64, 1.22) |
| Upper urinary tract rate | Baseline | 1.43 (0.97, 2.10) | 1.30 (0.90, 1.86) | 1.10 (0.72, 1.67) |
|  | Follow up 1 | **0.61 (0.41, 0.90)** | 1.01 (0.68, 1.49) | **0.61 (0.39, 0.95)** |
|  | Follow up 2 | **0.38 (0.18, 0.77)** | 0.61 (0.30, 1.15) | 0.63 (0.26, 1.50) |
|  |  |  |  |  |
| *Ultrasound: urinary bladder* | |  |  |  |
| Distorted bladder shape | Baseline | **0.22 (0.13, 0.36)** | **0.48 (0.32, 0.73)** | **0.46 (0.25, 0.81)** |
|  | Follow up 1 | **0.29 (0.16, 0.52)** | **0.46 (0.25, 0.81)** | 0.64 (0.32, 1.26) |
|  | Follow up 2 | 0.43 (0.10, 1.64) | 0.61 (0.14, 2.33) | 0.70 (0.13, 3.87) |
| Irregular bladder wall | Baseline | **0.36 (0.24, 0.53)** | **0.52 (0.35, 0.75)** | 0.70 (0.43, 1.12) |
|  | Follow up 1 | **0.41 (0.27, 0.61)** | 0.87 (0.61, 1.24) | **0.47 (0.31, 0.71)** |
|  | Follow up 2 | **0.49 (0.31, 0.77)** | **0.37 (0.20, 0.63)** | 1.35 (0.73, 2.56) |
| Any bladder masses | Baseline | **0.40 (0.26, 0.60)** | **0.62 (0.41, 0.94)** | 0.64 (0.39, 1.06) |
|  | Follow up 1 | **0.20 (0.13, 0.29)** | **0.34 (0.23, 0.48)** | **0.59 (0.37, 0.93)** |
|  | Follow up 2 | **0.31 (0.18, 0.50)** | **0.40 (0.22, 0.69)** | 0.77 (0.38, 1.58) |
| Any pseudopolyps | Baseline | **0.35 (0.14, 0.76)** | **0.30 (0.11, 0.69)** | 1.18 (0.38, 3.79) |
|  | Follow up 1 | **0.20 (0.08, 0.43)** | **0.39 (0.18, 0.79)** | 0.51 (0.18, 1.34) |
|  | Follow up 2 | **0.00 (0.00, 0.16)** | **0.15 (0.01, 0.75)** | 0.02 (0.00, 2.32) |
| Bladder wall thickening | Baseline | **0.24 (0.18, 0.32)** | **0.48 (0.36, 0.63)** | **0.50 (0.35, 0.70)** |
|  | Follow up 1 | **0.28 (0.22, 0.37)** | **0.64 (0.50, 0.81)** | **0.45 (0.33, 0.59)** |
|  | Follow up 2 | **0.27 (0.18, 0.39)** | **0.25 (0.15, 0.40)** | 1.05 (0.60, 1.89) |
|  |  |  |  |  |
| *Ultrasound: upper urinary tract* | |  |  |  |
| Dilated left or right | Baseline | 0.69 (0.38, 1.24) | 0.72 (0.43, 1.17) | 0.97 (0.49, 1.87) |
| pelvis | Follow up 1 | 0.62 (0.35, 1.09) | 0.63 (0.34, 1.12) | 1.00 (0.51, 1.97) |
|  | Follow up 2 | 0.88 (0.28, 2.47) | 0.90 (0.25, 2.72) | 0.98 (0.24, 4.11) |
| Dilated left or right | Baseline | 1.10 (0.63, 1.93) | 0.67 (0.36, 1.19) | 1.65 (0.84, 3.31) |
| ureter | Follow up 1 | 0.69 (0.36, 1.31) | 0.80 (0.42, 1.51) | 0.86 (0.41, 1.81) |
|  | Follow up 2 | **0.27 (0.06, 0.85)** | **0.01 (0.00, 0.28)** | ***** |

* The odds ratio for this effect was highly variable due to the prevalence at both surveys being close to zero. We chose to omit this effect due to its uncertainty.

## Supplementary Table 3. Sample sizes of schools and participants in *S. mansoni* analyses. Frequencies are broken down by morbidity and heavy-intensity prevalence category. Cells contain the number of schools with the number of participants in parentheses. Participants are school-age children, with morbidity and infection data, aged 6-15 years, and enrolled between 2003 and 2008.

| Morbidity | Survey | Heavy-intensity prevalence < 1% | Heavy-intensity prevalence ≥ 1% and < 5% | Heavy-intensity prevalence ≥ 5% |
| --- | --- | --- | --- | --- |
|  |  |  |  |  |
| *Ultrasound* |  |  |  |  |
| Image pattern | Baseline | 24 (3225) | 5 (429) | 16 (1423) |
|  | Follow up 1 | 22 (3889) | 7 (537) | 12 (959) |
|  | Follow up 2 | 24 (3631) | 6 (434) | 7 (454) |
| Enlarged portal vein | Baseline | 24 (3164) | 5 (464) | 16 (1501) |
|  | Follow up 1 | 19 (3296) | 7 (537) | 12 (960) |
|  | Follow up 2 | 24 (3637) | 6 (435) | 7 (456) |
|  |  |  |  |  |
| *Laboratory* |  |  |  |  |
| Lab-confirmed blood in | Baseline | 57 (8080) | 17 (2421) | 22 (2718) |
| stool | Follow up 1 | 50 (8166) | 12 (1400) | 17 (1871) |
|  | Follow up 2 | 26 (4731) | 6 (709) | 8 (776) |
| Lab-confirmed diarrhea | Baseline | 46 (6290) | 17 (2418) | 22 (2715) |
|  | Follow up 1 | 42 (5537) | 12 (1400) | 17 (1858) |
|  | Follow up 2 | 18 (2213) | 7 (847) | 9 (815) |
|  |  |  |  |  |
| *Self-report* |  |  |  |  |
| Self-reported diarrhea | Baseline | 65 (8890) | 20 (2627) | 29 (3319) |
|  | Follow up 1 | 50 (8111) | 12 (1368) | 17 (1840) |
|  | Follow up 2 | 36 (5355) | 8 (890) | 11 (946) |
| Abdominal pain | Baseline | 35 (4457) | 15 (1685) | 25 (2724) |
|  | Follow up 1 | 24 (4597) | 9 (986) | 13 (1365) |
|  | Follow up 2 | 36 (5349) | 8 (889) | 11 (946) |

## Supplementary Table 4. Odds ratios and 95% credible intervals from Bayesian logistic regression models comparing morbidity positive proportions between heavy-intensity prevalence categories within surveys for S. mansoni-related morbidities not presented in Table 2. Bold font indicates the 95% credible interval does not contain one. Participants are school-age children, aged 5-15 years, and enrolled between 2003 and 2008. Corresponding plots of unmodeled morbidity prevalence are in Figure 3 and Supplementary Figure 4.

| Morbidity | Survey | < 1% vs. ≥ 5% | 1-5% v. ≥ 5% | < 1% v. 1-5% |
| --- | --- | --- | --- | --- |
|  |  |  |  |  |
| *Ultrasound* |  |  |  |  |
| Image pattern B-F | Baseline | **0.37 (0.27, 0.50)** | **0.50 (0.28, 0.84)** | 0.75 (0.43, 1.34) |
|  | Follow up 1 | **0.43 (0.31, 0.61)** | 1.44 (0.98, 2.12) | **0.30 (0.20, 0.45)** |
|  | Follow up 2 | 1.54 (0.89, 2.86) | 0.79 (0.38, 1.64) | **1.96 (1.16, 3.50)** |
|  |  |  |  |  |
| *Laboratory* |  |  |  |  |
| Lab-confirmed blood in | Baseline | **0.23 (0.11, 0.47)** | **0.34 (0.14, 0.75)** | 0.68 (0.28, 1.75) |
| stool | Follow up 1 | **0.32 (0.15, 0.69)** | 0.85 (0.34, 2.01) | **0.37 (0.16, 0.95)** |
|  | Follow up 2 | **0.12 (0.02, 0.52)** | 0.25 (0.02, 1.52) | 0.49 (0.06, 7.39) |
|  |  |  |  |  |
| *Self-report* |  |  |  |  |
| Abdominal pain | Baseline | **0.73 (0.64, 0.84)** | 0.92 (0.78, 1.08) | 0.80 (0.69, 0.93) |
|  | Follow up 1 | 1.04 (0.88, 1.23) | 0.83 (0.68, 1.01) | **1.26 (1.05, 1.51)** |
|  | Follow up 2 | 1.07 (0.90, 1.28) | **0.77 (0.62, 0.95)** | **1.40 (1.17, 1.67)** |

* The odds ratio for this effect was highly variable due to the prevalence at both surveys being close to zero. We chose to omit this effect due to its uncertainty.

# Bibliography

1. Gelman A, Jakulin A, Pittau MG, Su Y-S. A weakly informative default prior distribution for logistic and other regression models. Ann Appl Stat. 2008;2(4):1360-83. doi: 10.1214/08-AOAS191.

2. Gelman A. Prior distributions for variance parameters in hierarchical models. Bayesian Anal. 2006;1(3):515-33.

3. Plummer M, editor. JAGS: A program for analysis of Bayesian graphical models using Gibbs sampling. Vienna, Austria: Austrian Science Foundation; 2003.

4. Plummer M, Best N, Cowles K, Vines K. CODA: convergence diagnosis and output analysis for MCMC. R news. 2006;6(1):7-11.

5. Plummer M. rjags: Bayesian Graphical Models using MCMC. R package version 4-10 ed2019.
